# Monitoring the apical growth characteristics of hairy roots using non‐invasive laser speckle contrast imaging

**DOI:** 10.1002/elsc.202100086

**Published:** 2021-12-13

**Authors:** Carolin Schott, Thomas Bley, Thomas Walter, Janis Brusius, Juliane Steingroewer

**Affiliations:** ^1^ Institute of Natural Materials Technology TU Dresden Bioprocess Engineering Dresden Germany; ^2^ Moor Instruments Ltd Axminster UK

**Keywords:** apical growth, hairy roots, laser speckle contrast analysis, non‐invasive, real‐time monitoring

## Abstract

Hairy roots are used to produce plant agents and additives. Due to their heterogeneous structure and growth characteristics, it is difficult to determine growth‐related parameters continuously and in real time. Laser speckle contrast analysis is widely used as a non‐destructive measurement technique in material testing or in medical technology. This type of analysis is based on the principle that moving objects or particles cause fluctuations in stochastic interference patterns known as speckle patterns. They are formed by the random backscattering of coherent laser light on an optically rough surface. A Laser Speckle Imager, which is well established for speckle studies of hemodynamics, was used for the first time for non‐invasive speckle measurements on hairy roots to study dynamic behavior in plant tissue. Based on speckle contrast, a specific flux value was defined to map the dynamic changes in the investigated tissue. Using this method, we were able to predict the formation of lateral strands and to identify the growth zone in the apical root region, as well as dividing it into functional regions. This makes it possible to monitor physiological processes in the apical growth zone in vivo and in real time without labeling the target structures.

**Abbreviation**
LSI: Laser Speckle Imager

## 1 INTRODUCTION

Hairy roots are filamentous plant tissue cultures that occur naturally as a symptom of hairy root disease, caused by the plant pathogen *Agrobacterium rhizogenes* (synonym: *Rhizobium rhizogenes* [1]) [2, 3, 4, 5].

Due to their relatively fast growth and attractive secondary metabolite formation, hairy roots are used as plant production platforms or as model objects for scientific research. Hairy roots are also characterized by simple, hormone‐free cultivation or high genetic, and metabolic stability [6, 7, 8, 9, 10].

The focus of scientific research and development is on studying the synthesis and regulation of biomass growth and the production of secondary metabolites as a means of developing efficient biotechnological production processes [8, 11, 12].

The growth characteristics of hairy roots are comparable to those of primary and adventitious roots of higher plants [13]. Hairy roots are mainly characterized by apical growth and the formation of lateral side strands. Apical growth is defined by cell division and cell elongation in the growth zone at the root tip [14, 15, 16, 17].

The functional areas in the apical growth zone are organized linearly, in proximal direction, into zones featuring cell division, elongation, root hair, and differentiation [17, 18, 19].

Due to the heterogeneous structure of hairy roots, determining biomass growth is challenging. Because of the morphological characteristics of hairy roots, representative sampling is not possible. As the direct determination of biomass requires the destruction of all the plant material, current investigations have to be parallelized and carried out with a high number of experimental approaches [11, 20, 21]. Experiments for screening and optimization are consequently very time‐consuming and cost‐intensive [11].

In order to shorten the times required for experimentation and the development of biotechnological processes, a continuous, direct measurement method for determining growth‐relevant characteristics is required. To identify physiological correlations, it is important to carry out the analyses non‐invasively and in real time.

Compared to electrochemical sensors, optical sensor systems operate non‐invasively and are attracting increasing attention in biotechnology [22, 23, 24].

The optical methods applied include NMR spectroscopy and fluorescence measurement or laser flow cytometry [23, 25]. In particular, flow cytometric analysis provides detailed intracellular insights into the components of single cells in cell populations [25].

Spectroscopic sensors are used for real‐time, in‐line monitoring to optimize productivity and ensure product quality in research and the pharmaceutical industry [26].

For example, great progress has been achieved in the real‐time monitoring of biofilms, especially in the development of fluorescence sensors [24, 27]. For an informative, continuous analysis of biofilms, an online‐capable fluorescence sensor has been developed that allows the non‐invasive measurement of important biological fluorophores in biofilms [27].

**PRACTICAL APPLICATION**

Hairy roots are used for the biotechnological production of natural agents. Due to their heterogeneous biomass structure, the continuous, non‐invasive real‐time determination of growth‐relevant parameters is difficult.

Laser speckle contrast analysis is a non‐destructive, fast means of measurement that is based on the evaluation of stochastic interference patterns, known as speckle patterns. Speckles are formed when an optically rough surface is irradiated with coherent light. Speckles contain a lot of relevant information about the illuminated object. The analysis of speckle contrast is based on the fact that dynamic motions in the object cause fluctuations in the induced speckle pattern.

This article shows that the laser speckle technique can be used as an innovative, non‐invasive method for analyzing filamentous structures in the field of biotechnology, such as hairy roots. Laser speckle contrast analysis was used to identify the apical growth zone and to predict the formation of side strands.

However, fluorescence spectroscopic analysis with plant material is limited. Staining plant cells with organic fluorescent dyes or labeling them with fluorescent marker proteins is difficult due to the persistence of the plant cell wall. In addition, interfering signals usually occur due to the autofluorescence of plant chromophores or the formation of secondary metabolites [28, 29].

One potential approach for measuring plant tissue is laser speckle imaging [30, 31]. Laser speckle imaging is an optical, non‐invasive method characterized by a simple experimental setup consisting of a laser source and a detector. Among other things, laser speckle imaging can be used to determine material properties such as hardness and porosity [30, 32–35].

Laser speckle imaging is based on the evaluation of stochastic interference patterns, known as speckle patterns. Patterns of this kind are formed by the random backscattering of coherent laser light on an optically rough surface. The speckle pattern contains an encoded three‐dimensional map of the surface structure. The specific statistical properties of the speckle patterns can be used to determine minimal changes to the surface and structural defects [32, 33, 36–38].

Fundamental methods for deriving the complex statistical properties of speckle patterns are discussed in detail by Goodman, 1975 [37].

When biological objects are irradiated with coherent light, backscattered fluctuating interference patterns called dynamic speckles or biospeckles are formed. The temporally or spatially resolved fluctuation of the dynamic speckle pattern – biospeckle activity – is caused by physical or biological processes affecting the illuminated object [33, 36, 39–44].

Biospeckle activity results from the change in the laser speckle contrast, which quantifies image blurring. The speckle contrast (*k*) is defined as the ratio of the standard deviation (σ) to the mean intensity (⟨I⟩)of a speckle pattern [36, 37, 45]:

(1) $$k\;=\frac{\sigma }{\left\langle I\right\rangle }$$

If the standard deviation of the intensity is low compared to the unchanged mean intensity, this leads to a reduction in the speckle contrast.

A low speckle contrast is thus caused by high blurring indicating the fluctuation of the speckles. High fluctuation is implied by dynamic behavior, such as patterns of movement in the illuminated particles in the object [37, 39, 46].

Biospeckle activity is influenced by Brownian molecular motion as well as physiological processes related to the movement of components [36, 40, 44]. In line with Zdunek et al., 2017 [44], these processes are classified into four main groups: (1) active movement of microorganisms, (2) changes in the shape of the cell organelles or tissues, (3) secondary transport processes of cells, particles, and fluids throughout the organism, and (4) transport processes of organelles and molecules within individual cells [40, 43, 44].

The statistical properties of the dynamic speckles can be used to obtain information about these processes and the object attributes [36, 40, 47, 48]. According to Goodman, 1975 [37] and Rabal and Braga, 2009 [36], the fluctuation intensity of time‐integrated dynamic speckle patterns can be determined using the intensity autocorrelation function [37, 49].

Successful technical implementations using the biospeckle phenomenon have been developed to measure perfusion processes in animal tissues [43, 50–52].

Speckle measurements have also been used to study other biological processes and phenomena, such as assessing the quality of fruits [44, 53–56], detecting the growth of fungi, bacteria, and parasites, studying the viability of plant seeds [57, 58], determining infections on leaves [48], and characterizing leaf and root growth [43, 50].

In most publications, the speckle contrast or the derived biospeckle activity was used as a cumulative parameter to describe dynamic behavior in the object structure [40, 47].

Individual process variables are difficult to interpret based on the speckle signal due to the overlapping and complexity of physical and biological processes.

Initial approaches to identify specific processes based on the speckle signal include studies on starch and chlorophyll accumulation in apples, thigmotropism in roots, cytoplasmic streaming, or water transfer in leader bundles [40, 50, 54, 55, 58].

Speckle analyses of plant structures published by Ansari et al., 2018 [48], detected areas of infection on leaves of *Plumeria rubra* and *Epipremnum aureum* from divergent speckle fluctuations.

Braga et al., 2009 [43] developed a simple system to determine biospeckle activity among in vitro root strands of *Coffee arabica* and *Eucalyptus grandis* shoot cultures in a solid culture medium. The roots could be clearly differentiated from the background noise on the basis of their higher speckle fluctuation, and it was demonstrated that biospeckle activity can be interpreted as an indicator of cellular molecular activities such as cytoplasmic streaming in root cells [43].

In studies by Ribeiro et al., 2014 [50] biospeckle activity was measured in the roots of *Zea mays*, *Jatropha curcas*, and *Citrus limonia*. From the results, it was possible to characterize biospeckle activity distribution along the root strands and changes in activity in response to root stimuli. The root apex was identified as a region with high biospeckle activity. The intensity decreased at a distance of 1–5 mm from the tip in a proximal direction. From these observations, the end of the root's elongation zone was estimated. The region with the highest biospeckle activity was assigned to the cell division region [50].

In previous speckle studies on hairy root networks, biospeckle activity was first shown to correlate with biomass growth [30, 31]. In Chen et al., 2019 [30] and Schott et al., 2020 [31], biospeckle activity was defined as the sum of all areas of the imaged hairy root network that featured dynamic shifts in the speckle pattern.

Speckle contrast was then used to differentiate between areas of high and low dynamic changes. As a result, with increasing culture age, significantly higher activity was observed in the apical root regions, allowing initial interpretations of the physiological state of the overall culture [31]. The technical setup placed limits on any further differentiation and interpretation.

For a more detailed investigation of apical root regions in hairy roots, the present work did not observe the whole hairy root network as described above, instead focusing on the analyses at single strand level.

To increase the spatial resolution and information density, the experimental parameters were optimized and the Laser Speckle Imager (LSI FLPI‐2) from Moor Instruments Ltd (Axminster, UK) was used for the first time to investigate hairy root tissues.

The LSI FLPI‐2 is a functional instrument that has been successfully used in tissue transplantation clinical trials to image blood microcirculation [59, 60, 61, 62]. The operating principle is based on laser speckle contrast analysis and was originally developed to determine perfusion dynamics [36, 51, 63, 64].

When the LSI was applied, specific apical growth dynamics were detected with high‐resolution signals.

## 2 MATERIALS AND METHODS

### 2.1 Sample preparation

Hairy roots of beetroot (*Beta vulgaris*, *B. vulgaris*) were used as a model organism as previously described in Schott et al., 2020 [31]. The cultures were induced by transformation with *A. rhizogenes* ATCC 15834 in line with the description by Pavlov et al., 2002 [65] and Weber et al., 2010 [69].

The speckle contrast analysis was performed on single hairy root strands. Each single strand was separated from a 14‐wk‐old culture with an initial length of 1.5 mm and placed in a standard 92‐mm‐width single‐use Petri dish (Sarstedt AG & Co. KG, Nümbrecht, Germany) on a solid Murashige and Skoog medium [67] (Duchefa Biochemistry B.V, Haarlem, The Netherlands) supplemented with 30 g/L sucrose (Duchefa Biochemistry B.V) and 5.5 g/L phyto agar (Duchefa Biochemistry B.V) [68, 69, 70, 71].

### 2.2 Laser speckle imaging

Speckle images were acquired using the moorFLPI‐2 Full‐Field Laser Perfusion Imager (LSI FLPI‐2) from Moor Instruments Ltd, Axminster, UK, whose basic operating principle has been described by O'Doherty et al., 2009 and others [59–62, 72, 73].

This device uses an infrared laser at a wavelength of 785 nm as a light source. The tissue is imaged with a charged coupled device camera and video data is captured by a PC. The LSI FLPI‐2 measures to a maximum penetration depth of approximately 1 mm [61, 74].

**FIGURE 1:**
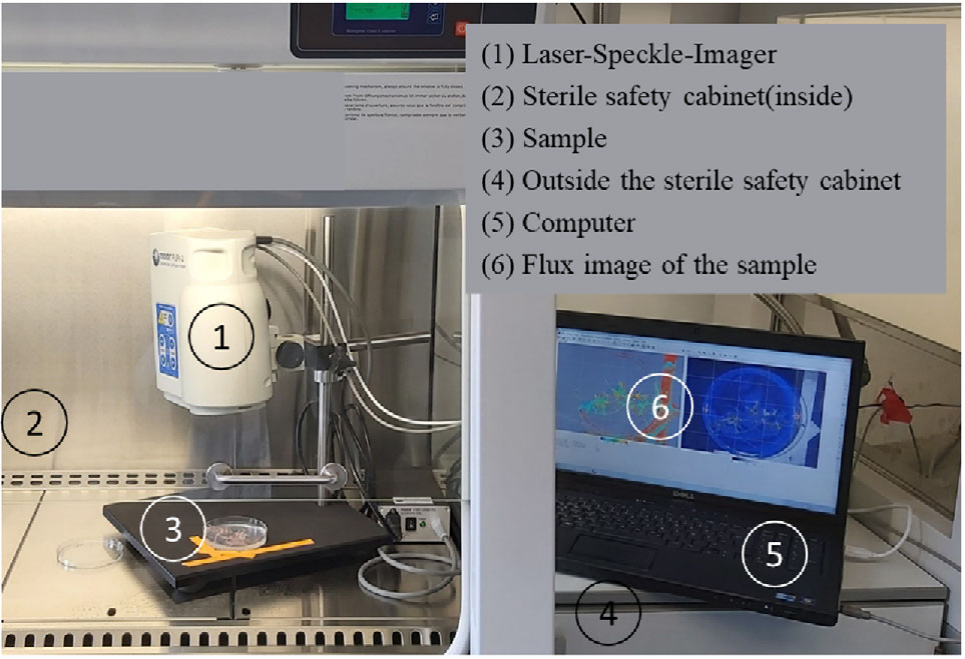
Laser Speckle Contrast Imager (LSI FLPI‐2), Moor Instruments Ltd, Axminster, UK), experimental set‐up for measuring plant tissue under sterile measurement conditions (Telstar safety cabinet, Azbil Telstar, S.L.U., Barcelona, Spain)

For sterile measurements of the individual hairy root strains, including in open cultivation dishes, the LSI FLPI‐2 was implemented in a safety cabinet (Telstar). To avoid interfering signals from the environment and the sample background, the experimental setup was covered during the measurements (Figure 1).

LSI‐FLPI‐2 measurement software V2.0 was used to control and configure the instrument and to record the data. The review software V 5.0 was used for image processing and analysis after the measurement [59, 60, 61, 62].

The LSI FLPI‐2 was attached above the sample at a working distance of 18.54 cm. For each sample and measurement, 16 image stacks consisting of 25 images were acquired with a frame rate of 25 Hz. The image stacks were recorded at time intervals of 2 s. For each measurement, a total of 400 images were acquired in 32 s with an exposure time of 20 ms (*Temporal processing* [59, 60, 61, 62]). The acquisition area of the speckle images was set to the size of the cultivation dish (92 mm standard Petri dish).

The image processing software generates color‐coded maps of tissue dynamics [61, 62, 74].

The color coding is done by the specific flux value which is derived from the correlation of the speckle contrast (Equation 1) and can be defined as follows.

(2) $$\mathrm{Flux}\propto {\left(\frac{\left\langle I\right\rangle }{\sigma }\right)}^{2}$$

### 2.3 Evaluation of the laser speckle images

Color‐coded flux images of the individual hairy root strands were further processed by the LSI FLPI‐2 review software V 5.0. To evaluate flux images from hairy roots, a custom algorithm was established.

**FIGURE 2:**
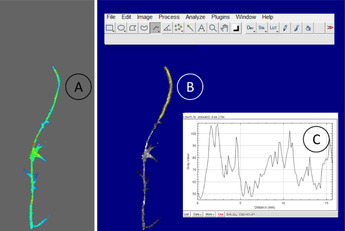
Evaluation of the laser speckle images, (A) flux image (color‐coded), (B) flux image transformed in gray‐scale image and selection of the ROI line, and (C) dynamic profile (gray value) as a function of the root length

An average flux image was calculated from the measured flux images for each sample and measurement time point (Figure 2A). The average flux value along the root strands was determined, along with the maximum and minimum flux values.

The flux image was transformed into a grayscale image to determine the dynamic profile (Figure 2B). The qualitative flux value profile was evaluated using Image J software [75]. After loading the grayscale image into Image J, the scale was first set according to the resolution of the speckle image (1 mm = 6.4383 px). An ROI line was drawn along the root strand in accordance with the root diameter, then evaluated as a grayscale profile as a function of the root length (Figure 2B,C).

## 3 RESULTS AND DISCUSSION

In the study described here, laser speckle contrast analysis was used to identify and determine physiological processes and growth‐related parameters of hairy roots at the single‐strand level. Speckle dynamics were labeled as a color‐coded flux value.

The flux value is a software‐specific dimensionless unit for the average perfusion dynamics in the tissue. Following Equation 2 and compared to Equation 1, the flux value is indirectly proportional to the speckle contrast. The perfusion dynamics or dynamic behavior mainly depend on the particle concentration and velocity, among other factors [61, 62, 74].

Dynamic behavior is a term for processes such as cell movements or particle oscillations that cause changes in the tissue. A high flux value corresponds to high dynamics. A low flux value symbolizes lower dynamics [36, 44, 61, 62, 74].

### 3.1 Evaluation of the flux profile of the entire hairy root strand

**FIGURE 3:**
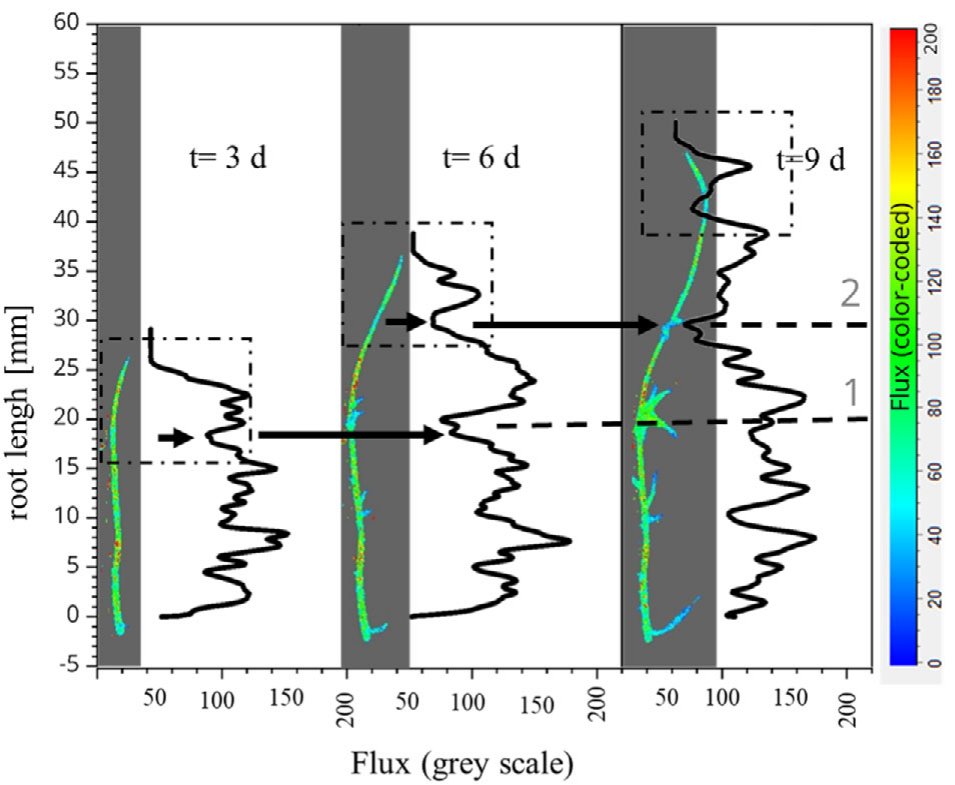
Dynamic behavior along the root length of a hairy root (*B. vulgaris*, 26°C, MS medium [solid]) at time *t* [d] with the Laser Speckle Imager: FLP‐2 (*λ* = 785 nm, *T*_exp_: 20 ms, frame rate: 25 Hz), colored zones: flux image/‐value, black line: flux profile (sliding average *n* = 8), dashed line: auxiliary line with 1, 2, and 3 for local minima, dashed square: apical region

Measurements of main root strands were generally presented with an average range of values from 0 to 200 flux, labeled from blue to red (Figure 3). Values above 200 flux were also shown in red. The flux value averaged over the entire root length was between 70 and 80 for all samples.

Due to the lack of data available to evaluate plant material with the LSI‐FLPI‐2, the absolute flux values could not be compared to flux values of other plant tissue structures.

Typical flux values measured with the LSI‐FLPI‐2 and the predecessor model vary between species and different tissues. In particular areas of the mouse cortex, flux values can range between 1000 and 3500 perfusion units, with typical baseline recordings of about 1500–2000 perfusion units [76, 77]. Baseline recordings in the human cortex are in a comparable range [72, 78]. Flux values of human skin microcirculation are in a range of 35–420 depending on the actual body part and the physiological state [62].

In comparison, the determined flux values for hairy roots are in a reasonable measurement range for this measurement system. Furthermore, a broad database for plant tissue needs to be compiled.

However, for all samples measured, the flux value along the root strand varied depending on the distance from the root tip (*d*_A_).

Based on the evaluation of the flux value profiles along the root strands, the following recurring characteristics were identified. First, by evaluating the flux value, the formation of lateral side strands was predicted based on a local flux minimum. Second, a recurrent pattern was observed in the apical root area for all measurement time points.

All the samples examined exhibited comparable characteristics with regard to the flux profile. In the following, the qualitative flux profile along a randomly selected sample is presented as a representative example of single strand analysis.

The main root strand of the sample (Figure 3) showed a total length growth of 29.4 mm during the cultivation period of 9 days and formed eight clearly observable lateral strands.

The formation of a side strand could be clearly identified from the flux value profile by the formation of a local minimum, e.g. in the cases of auxiliary lines 1, 2, and 3 in Figure 3. For example, at cultivation time *t* = 3 days, the branching point marked by auxiliary line 1 was already characterized by a local minimum. At this time, a lateral root was not seen to sprout on the main root; it was formed visibly later. It is known that the lateral root forms endogenously behind the apical growth zone from the central tissue of the main root [17, 79, 80].

The decreasing flux value was attributed to the reduction of cell movement as a result of differentiation processes, as well as a decrease in the cell and particle concentration inside the tissue. A change in the fluid flow in the central tissue or the modification of the root structure can also be considered as a source of flux variations. In addition, biochemical processes due to cell differentiation influence the interaction between the laser and the tissue. On the other hand, the thickening or overlapping of cell layers may have reduced the penetration depth of the laser, so that dynamic processes in deeper tissue layers were not visualized.

The location of the branching point is important for the differentiation of the root growth zone. Branching points at hairy roots indicate cell differentiation and are stationary [81]. Accordingly, it can be assumed that the transition from the elongation zone to the differentiation zone was positioned through the first branching point located in the proximal direction and that, consequently, the growth zone must have been located between the root apex and the identified branching point [17–19, 79, 80].

The growth zone of hairy roots is located in the apical root area between the first differentiation point and the root apex. The growth dynamics in the apical root zone depend mainly on the cell division rate and cell elongation [18, 19, 82].

The flux profile in the apical root regions showed a recurrent pattern characterized by the formation of a local maximum (Figure 3, dashed square). The evaluation of the flow profile in the apical root region is discussed in detail below.

### 3.2 Differentiation of the apical growth zone based on the flux profile

Apical root growth is characterized by the physiological processes in the growth zone. The apical root region in the proximal direction is divided into (i) root cap, (ii) cell division zone, (iii) elongation zone, (iv) root hair zone, and (v) differentiation zone [17, 83].

The growth zone includes the area from the root cap to the end of the elongation zone. The size of the growth zones and their intrinsic distribution depend on the particular growth state of the root [17, 18, 79].

**FIGURE 4:**
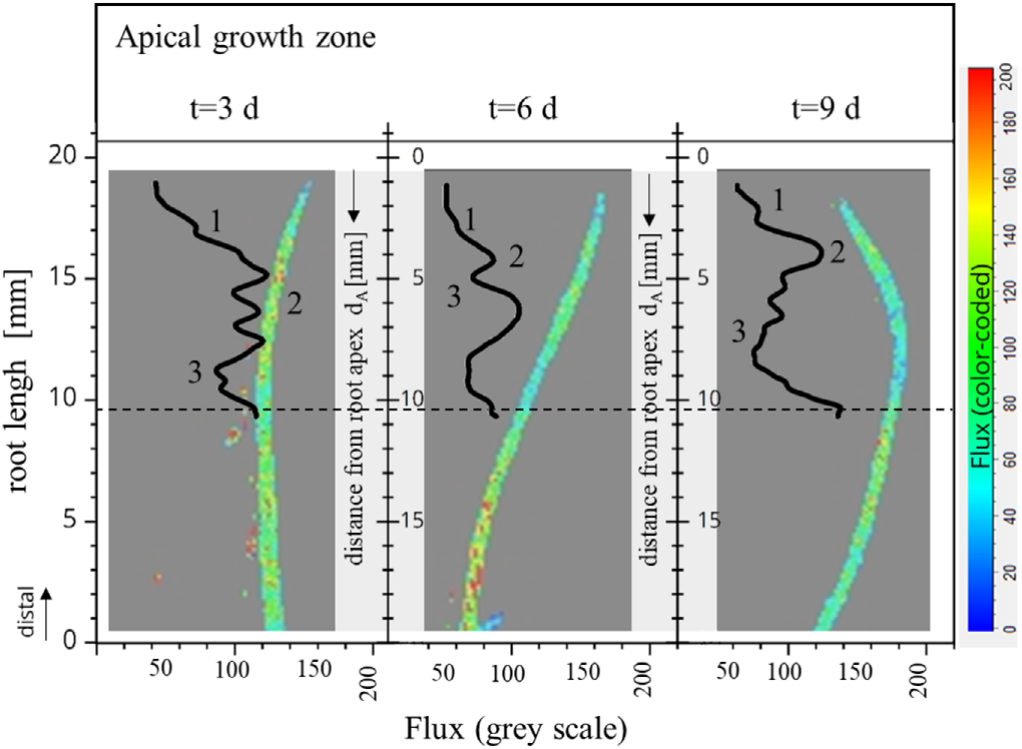
Analysis of apical hairy root tissue (*B. vulgaris*, 26°C, MS medium [solid]) at time *t* [d] using the Laser Speckle Imager FLPI‐2 (*λ* = 785 nm, *T*_exp_: 20 ms, frame rate: 25 Hz), colored zones: flux image/‐value, black line: flux value (sliding average *n* = 8), distance from root apex (*d*_A_), dashed line: auxiliary line for *d*_A_ = 10 mm, 1, 2, and 3 marker points for local minima and maxima

The apical root zones of the randomly selected sample at the cultivation times *t* = 3, 6, and 9 days are highlighted in Figure 4. In Figure 4, the distance from the root apex (*d*_A_) was inserted as an additional y ordinate. From the flux profile of the apical root zones, a recurring pattern was determined in an evaluation range of *d*_A_ < 10 mm (Figure 4). The characteristic shapes are marked 1, 2, and 3 in Figure 4.

At the root tip for *d*_A_ = 0 to about *d*_A_ = 1–2 mm (Figure 4, 1), a small increase in the flux value was initially registered. Depending on the sample, a peak or shoulder was indicated. Subsequently, the flux value increased to a local maximum or maximal plateau (Figure 4, 2). However, it was not possible to determine a fixed distance value from the root peak for this area. The distance of the local intensity maximum from the root tip was different for each root strand.

For the evaluation, it was crucial that after reaching the maximum value or the maximal plateau, a decrease in the flux value to a local minimum was detected (Figure 4, 3).

As previously explained, a local minimum indicates the formation of a side strand. Based on this result, a differentiation region was defined, as shown in Figure 4, 3. Based on the localization of the differentiation zone, the end of the elongation zone was determined.

Consequently, the section with higher flux values (Figure 4, 2) and thus higher dynamics could be assigned to the elongation zone.

In line with the description of the linear organization of the apical growth zone at the beginning of this section [19, 79, 82, 84, 85], the cell division zone is located in the proximal direction after the elongation zone. The area of the marker placed in Figure 4, 1 with an extension of approximately 1–2 mm at the root tip was therefore assigned to the cell division zone.

The cell division zone is enclosed by the root cap at the root tip [17, 79]. No further differentiation between the root cap and the crossover into the cell division zone was identified based on the flux profile.

New cells are constantly formed in the cell division zone. The generated cells are attached distally to form the root cap and released proximally into the elongation zone [17, 80]. The dynamic behavior in the root tissue resulting from these processes causes the flux value to increase until approximately 1–2 mm in front of the root apex. This is comparable with Sacks et al., 1997 [84] and Ribeiro et al., 2014 [50], where the end of the cell division zone in maize roots was observed to be approximately 2 mm before the root tip.

However, it should be noted that the length of the cell division zone is not always the same. In addition to the proliferation rate, the length of the cell division zone is also influenced by the residence time of the cells in the meristem [18, 19, 86].

The cells that are transferred proximally from the meristem into the elongation zone, pass through it in only a few seconds and can be elongated up to 10 times their length [80]. The dynamic cell movement in the root tissue can cause an increase in the flux value (Figure 2, 4).

The spatio‐temporal dynamics of plant root elongation growth are most commonly quantified using the relative elemental growth rate (REGR) [87] as a function of distance from the root apex [18, 19].

The spatially and temporally resolved REGR provides a quantitative description of the size and distribution of functional areas in the growth zone [82, 85].

The spatial distribution of REGR increases from zero at the meristematic tip to a maximum REGR_max_ in the elongation zone, then decreases back to zero at the end of the elongation zone [18, 19, 85]. Comparing this to the measured flux values, the course of the REGR can be traced and the maximum can be assigned to the elongation zone.

The spatial distribution of the flux values shows a comparable pattern. Assuming that the maximum dynamic behavior in the apical tissue structure is indicated by the maximum REGR, the assignment of the elongation zone was unambiguously based on the maximum flux value (Figure 2, 4).

The length of the elongation zone was estimated for the individual root sample based on the indicated differentiation point, and was different for all samples. The identified elongation zones were located in a range of about 1–2 mm < *d*_A _< 10 mm in front of the root apex (Figure 4). For example, in an intact maize root, the elongation zone began approximately 1–2 mm in front of the root apex, with a total growth zone length of 9–10 mm [19].

The length of the elongation zone depends on the number of cells transferred into the elongation zone over time. Transfer processes, diffusion, and reaction rates are also influenced by physical parameters such as the temperature, concentration gradients, or the cell turgor [19, 79, 80, 82, 86].

In summary, based on the results presented in Figures 3 and 4, it was possible to identify a characteristic profile of the flux value along the apical root region for each sample. Furthermore, in relation to the predominant apical growth dynamics of the hairy roots, the differentiation of the growth zone was performed based on the qualitative flux value. The maximum flux value in the growth zone was assigned to the elongation zone, and the end of the elongation zone was characterized by a decrease in the flux value to a local minimum.

The classical techniques for analyzing the distribution of elongation growth on the root usually require the labeling of the rhizodermis. Analyses with high spatial and temporal resolution are time‐consuming, which limits the general application of these techniques [19].

Therefore, advances in digital image processing are now widely used for automated analysis [19, 84, 88]. With digital image analysis, it is possible to track surface changes on the rhizodermis to modulate the REGR [19, 66, 89].

However, the laser speckle technique does not only analyze dynamic changes at the surface: this innovative measurement technique also provides information about the tissue interior. Laser speckle contrast analysis offers the potential to non‐destructively measure and image tissue‐internal or intracellular physiological processes, such as growth changes during water scarcity, in real time.

These investigations of plant hairy root cultures with the LSI‐FLPI2 have successfully shown that the dynamic behavior along the root strand follows a characteristic pattern. This characteristic pattern was in part assigned to physiological processes such as cell elongation or cell differentiation. Using the same procedure, the results were also reproduced with single hairy root strands of *Datura stramonium* (data not shown).

To establish the laser optical methods and to optimize the measurement application used to evaluate plant physiological processes, a broad database is necessary.

Due to the high complexity and overlapping of plant physiological processes, it is a challenge to identify individual dynamics [17, 79].

Based on the results of the speckle measurements performed in this work, a platform was generated for further work on developing a fast, non‐invasive measurement method to elucidate the physiological relationships of root growth. The main advantage of this method is that the speckle measurement was performed in vivo, without external and internal markers.

## 4 CONCLUDING REMARKS

In the work presented here, the LSI FLPI‐2 was used for the first time to investigate hairy roots. Dynamic behaviors in the object were mapped using the device‐specific flux value, averaged over the root's longitudinal cross‐section.

From the flux values obtained, it was possible to identify recurring characteristic patterns along the root strand. The formation of lateral strands at the main root was predicted and the apical growth zone was localized. Furthermore, within the growth zone, the area of the elongation zone was clearly determined based on the flux value.

It was successfully demonstrated that laser speckle contrast analysis can be used as a rapid, non‐invasive measurement method for directly detecting dynamic growth behavior in hairy roots on a solid culture medium.

Another possible application of the presented laser speckle contrast analysis was demonstrated in examinations of non‐sterile plant tissue (a thin slice of beet *B. vulgaris*) where fungal contamination had occurred. The infected tissue could be detected at an early stage from a decrease in the flux value, even before fungal contamination was visible (data not shown). Similar results were obtained by Ansari et al., 2018 [48] using laser speckle contrast analysis on infection sites on plant leaves, or in studies by Braga et al., 2005 [57] on the detection of fungi on bean seeds. Basically, the laser speckle monitoring method is not object‐specific and is characterized by a simple basic setup, which is why it can be transferred to a wide range of further biotechnological applications [36, 43, 46].

## NOMENCLATURE

**Table:** 

*A*	[px]	Area
*D*	[mm]	Distance
Flux	[–]	Flux parameter related to the product of average speed and concentration of moving particles
*k*	[–]	Speckle contrast
*I*	[mW]	Laser intensity
*m*	[mg]	Mass
*t*	[d]	Time
*T*	[ms]	Exposure time
Greek symbols		
*σ*	[–]	Standard deviation
*μ*	[d^–1^]	Specific growth rate
*μ*_L_	[d^–1^]	Specific elongation rate
Indices		
max		Maximum

## CONFLICT OF INTEREST

The authors have declared no conflict of interest.

## Data Availability

The data that support the findings of this study are available from the corresponding author upon reasonable request.
